# 

**Published:** 1886-05

**Authors:** 


DEVOTED TO

### DENTAL AND ORAL SCIENCE.

**W. C. BARRETT, M. D., D. D. S.,**

EDITOR.

THE NEW YORK DENTAL JOURNAL ASSOCIATION,

PUBLISHERS,

No. 35 West 46th Street, New York.

AND

No. 208 Franklin St., Buffalo, N. Y.

**$2.50 Per Annum in Advance, . . . . Single Copies, 25 Cents.**

Entered at the Post-Office, Buffalo, N. Y., as Second-Class matter.

